# Elevational Gradient of Vascular Plant Species Richness and Endemism in Crete – The Effect of Post-Isolation Mountain Uplift on a Continental Island System

**DOI:** 10.1371/journal.pone.0059425

**Published:** 2013-03-12

**Authors:** Panayiotis Trigas, Maria Panitsa, Spyros Tsiftsis

**Affiliations:** 1 Department of Agricultural Biotechnology, Agricultural University of Athens, Athens, Greece; 2 Department of Environmental and Natural Resources Management, University of Ioannina, Agrinio, Greece; 3 Department of Botany, School of Biology, Aristotle University of Thessaloniki, Thessaloniki, Greece; Field Museum of Natural History, United States of America

## Abstract

Understanding diversity patterns along environmental gradients and their underlying mechanisms is a major topic in current biodiversity research. In this study, we investigate for the first time elevational patterns of vascular plant species richness and endemism on a long-isolated continental island (Crete) that has experienced extensive post-isolation mountain uplift. We used all available data on distribution and elevational ranges of the Cretan plants to interpolate their presence between minimum and maximum elevations in 100-m elevational intervals, along the entire elevational gradient of Crete (0–2400 m). We evaluate the influence of elevation, area, mid-domain effect, elevational Rapoport effect and the post-isolation mountain uplift on plant species richness and endemism elevational patterns. Furthermore, we test the influence of the island condition and the post-isolation mountain uplift to the elevational range sizes of the Cretan plants, using the Peloponnese as a continental control area. Total species richness monotonically decreases with increasing elevation, while endemic species richness has a unimodal response to elevation showing a peak at mid-elevation intervals. Area alone explains a significant amount of variation in species richness along the elevational gradient. Mid-domain effect is not the underlying mechanism of the elevational gradient of plant species richness in Crete, and Rapoport's rule only partly explains the observed patterns. Our results are largely congruent with the post-isolation uplift of the Cretan mountains and their colonization mainly by the available lowland vascular plant species, as high-elevation specialists are almost lacking from the Cretan flora. The increase in the proportion of Cretan endemics with increasing elevation can only be regarded as a result of diversification processes towards Cretan mountains (especially mid-elevation areas), supported by elevation-driven ecological isolation. Cretan plants have experienced elevational range expansion compared to the continental control area, as a result of ecological release triggered by increased species impoverishment with increasing elevation.

## Introduction

Elevational gradients are ideally suited for examining biodiversity drivers, as elevation is correlated with several environmental variables while providing more constant ecological conditions and history than can be obtained along continental or global spatial gradients and at the same time they represent reproducible environmental gradients that can be replicated across the globe [1], [2]. Two main patterns of species richness-elevation relationships are dominant in the relevant literature, i.e. decreasing and hump-shaped [3]. However, growing evidence suggests that the hump-shaped pattern, with diversity peaking at mid-elevations, represents half of all case studies for a wide range of taxa; the other half of the studies includes monotonic decreases or increases as well as roughly constant relations [2], [4], [5]. The proposed drivers of elevational gradients of biodiversity could be grouped into four main categories: (1) climatic variables like temperature and rainfall that determine energy availability and ecosystem productivity, (2) spatial aspects like area size and geometric constraints, i.e. mid-domain effect (MDE), (3) evolutionary history like clade age, speciation and extinction rates, and (4) biotic processes like competition, mutualism and ecotone effects [5]. Rapoport's latitudinal rule (i.e. the negative effect of latitude on species richness and its positive effect on species latitudinal range) has been extended to elevational gradients [6], [7] and has been subject of criticism and debate in different studies [8]–[12]. Elevational gradients of diversity are often taxon-specific and they depend on the geographical location of the gradient [4].

In contrast to elevational gradients of species richness, elevational gradients of endemism have attracted less attention. Analogous to species richness-elevation relationships, two main patterns of endemic species richness-elevation relationships have been documented, i.e. increasing [13]–[15] and unimodal [11], [16], [17]. The increase of endemism with elevation has been attributed to the increased isolation of higher elevations. Isolation promotes speciation and if the high mountain areas are large enough to allow population persistence and divergence, they may be rich in endemic elements [3], [18], [19]. However, the occasionally observed decrease of endemism at highest elevations has been explained by recent mountain uplifts providing too little time for speciation [15], [20], or by Pleistocene glaciations that might have led to the extinction of alpine endemics [11], [15], [20].

Habitat expansion (i.e. the larger spectrum of habitat types occupied by a species on islands than on the mainland) is a commonly recognized feature of the so-called ‘island syndrome’ [21]. This expansion has been explained either by restricted dispersal, intraspecific spillover resulting from density inflation, benign and predictable climate, or by the ecological release that results from species impoverishment [22]. Species elevational range is directly associated to species niche breadth. Increased elevational range involves increased physiological tolerance and habitat flexibility, as well as coexistence ability with different species. A study on elevational gradient of island plant species richness must value the influence of possible elevational range expansion of the whole flora, or of certain plant groups, in order to evaluate the observed patterns. Empirical observations about island elevational expansion have been mainly concerned with animals, e.g. [22]–[24] and, to our knowledge this research questions has not been previously tested on plants.

Elevational gradients of vascular plant species richness and endemism on Mediterranean islands have, to our knowledge, not been studied before. Previous studies have mainly been focused on the large mountain massifs of the world [11], [12], [14], [15], [17], [25]–[30] and on tropical islands [31]–[35]. Moreover, the combined effect of island and mountain isolation in the formation of elevational gradient patterns of species richness and endemism of island biota has received little attention. Increased ecological isolation with increasing elevation, however, has been linked to increased island endemism with altitude due to speciation processes on oceanic islands [36]. We used all available data on elevational ranges of vascular plant species to investigate species richness patterns along the entire elevational gradient of Crete. This is the first effort to study elevation gradient of an entire Mediterranean island flora interpolating species presence between known elevational range limits. This method, although weaker than direct field studies, has been commonly used to describe elevation gradients of species richness and endemism [11], [12], [15], [37], [38].

Area is a principal factor in all species richness analyses [39] and it usually declines with increasing elevation [19]. The available area for plants on mountains, however, may not be constant through time. Recent mountain uplift could directly affect the available area at high elevation over time, controlling species richness as well as elevation-driven ecological isolation and thereby the extent of endemism. The well known paleographic and geodynamic evolution of Crete offer a unique opportunity to investigate the influence of historical processes to the formation of the present patterns. A key feature for the interpretation of the elevational patterns of species richness and endemism on Crete is the recent uplift of the Cretan mountains. The mountains of Crete have a rather short history dating back to early Pliocene [40]–[42]. Since then, an uplift of c. 2000 m has been estimated and it was achieved mostly during the Pleistocene [42]–[44]. Thus, during the isolation period (early Pliocene) the height of the Cretan mountains scarcely exceeded 500 m and the elevational zones above 1500 m were formed during Pleistocene. Pollen records indicate that in the period of early Pliocene, the Mediterranean climate was warmer and wetter than it is today [45], [46]. Furthermore, late Miocene leaf assemblages from western Crete also indicate a warm-humid climate during this period [47]. As a result, a flora mainly composed of lowland species is expected to occupy the Cretan area at the isolation period and the already existing lowland species were the only available to occupy the subsequently uplifted mountain areas.

In this study, we examine the elevational gradient of vascular plant diversity on Crete, one of the tallest continental islands of the world. In particular, our aims are to document, describe, and explain the patterns of vascular plant species richness and endemism along the entire elevational gradient of Crete (0–2400 m). First, we describe the patterns along the elevational gradient. Then, we evaluate the drivers behind these patterns, focusing on elevation, area, geometric constraints (MDE), Stevens' elevational Rapoport effect and the post-isolation uplift of the Cretan mountains. Finally, we test the influence of the island condition and the post-isolation uplift of the Cretan mountains to the elevational range sizes of the Cretan plants, using a continental control area (Peloponnese).

## Methods

### Study area

**Figure 1:**
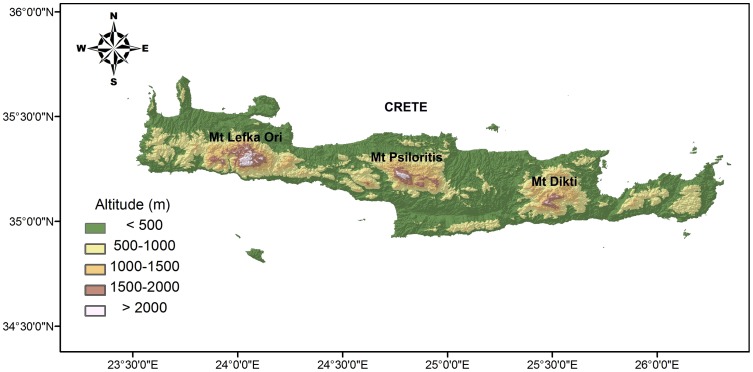
Topographic map of Crete with the three main mountain massifs.

Crete is the fifth largest island of the Mediterranean Basin and represents an important biodiversity hotspot especially due to its endemic vascular flora [48]. Its surface area covers 8265 km^2^ and it is situated in the centre of the south Aegean island arc, which also includes from East to West Rodos, Karpathos, Kasos, Antikithira and Kithira islands. The island is characterized by a mountainous terrain that forms moderate slopes and coastal planes in the north, southern slopes being much steeper forming lots of gorges and extensive cliff systems. Three main mountain ranges exist on Crete: Lefka Ori in western Crete (2452 m), with 57 summits over 2000 m, Psiloritis (2456 m) in the central part and Dikti mountains (2148 m) in eastern Crete (Fig. 1).

Crete represents, in the terminology of [49], a ‘subcontinental system’ in which most taxa were formerly part of a much larger continent, and therefore have evolved from a balanced continental flora. There is strong evidence that most Cretan endemic plant species and several non-endemics are remnants of this older continental flora and endemism is probably driven by loss of species on the mainland after island isolation [49], [50]. However, several cases of *in situ* radiation after island isolation have been suggested, especially of mountain ecotypes from lowland species [49]. Regarding animal taxa, both *in situ* evolution and endemism generated by extinction have been documented on Crete [51]–[54]. The contribution of long-distance dispersal to the formation of the present Cretan flora seems to be limited [50], [55]. An important exception includes plant species transferred to the island through human activities. Human presence on Crete lasts for over 9000 years, greatly affecting the flora and the vegetation of the island [56], [57]. It is noteworthy that the earliest archaeological evidence for Neolithic economies in SE Europe dates to c. 7000 cal BC, with the founding of a fully-fledged farming community at Knossos on the island of Crete [58]. It has been estimated that approximately one third of the wild flora of Crete has been introduced by man [49], [59], [60]. Moreover, human pressure and overgrazing has probably altered the present distribution patterns of many plant species [56], [61].

The palaeogeography of the area is rather complicated. The formation of the south Aegean island arc dates back to the middle Miocene [62]. The isolation of Crete from the neighbouring continental areas (Greek mainland and Asia Minor) had started at c. 10 Mya and at least since 5.3 Mya (early Pliocene) Crete is fully isolated. Crete is longer isolated than many oceanic islands, e.g. the current high islands of the Hawaiian archipelago range in age from 5.1 to 0.5 My [63], while the Canary Islands from 20 to 1.1 My [64]. For a detailed account of the palaeogeography of Crete see [65]–[67].

The neighboring peninsula of Peloponnese has been selected as a continental control area, in order to test possible elevational range expansions of Cretan plants, as Crete and the Peloponnese have equal elevational gradients (0–2400 m). Although evidently larger, Peloponnese shows significant topographic, climatic and geological similarities to Crete (Table 1). It is the southern peninsular part of Greece connected to the mainland by the narrow Isthmus of Corinth. It hosts a well-balanced continental flora, as the Corinthian gulf, which separates Peloponnese from the mainland, was formed during Pleistocene and represents only a weak biogeographical barrier [68].

**Table 1:** Physical and environmental characteristics of Crete and the Peloponnese.

Characteristic	Crete	Peloponnese
Area (km^2^)	8265	21076
Percent lowland (<500 m above sea level)	64.4	51.1
Percent highland (>1000 m above sea level)	11.7	16.3
Surface area of highland (km^2^)	966.2	3445.8
Highest peak altitude (m)	2456	2407
Latitude span (°N)	34°55′–35°41′	36°23′–38°20′
Substrate	mainly calcareous	mainly calcareous
Mean annual temperature at sea level (°C)	19.1 (18.5–19.6)	17.9 (16.8–18.6)
Mean annual rainfall at sea level (mm)	508 (298–662)	621 (410–921)

### Floristic diversity data sources

The Cretan flora has been intensively studied and an enormous amount of data concerning the distribution of vascular plants on the island is now available. To describe species richness patterns along the elevation gradient of Crete we used data on species elevation ranges found in literature, e.g. [69]–[73], herbarium specimens, data from the Flora Hellenica Database and unpublished data kindly offered to us by Nicholas Turland. The dataset includes minimum and maximum elevation limits for all species and subspecies registered (the term “species” is used hereafter for both species and subspecies for simplicity). Plant species elevational data have been collected for the entire elevational gradient of Crete, since incomplete sampling of environmental gradients can directly bias the resulting pattern. For example, a mid-elevation peak trend for a whole mountain will appear to be a decreasing or low plateau if only the upper half of the gradient is sampled [4], [5], [74]. Floristic data concerning the offshore islands and islets of Crete are not included in this study.

An important distortional factor in all island biogeography studies is the number of human-introduced species. Especially for islands with a long period of human habitation there will always be uncertainties whether the occurring species have been introduced by man, and whether the activities of humans have led to the extinction of native species [75], [76]. Plant species already introduced in prehistoric or early historic times may be perfectly integrated into the native plant communities and it may be extremely difficult, or even impossible, to distinguish them from the truly native ones [59]. The evidently human-introduced flora of Crete includes c. 245 alien species [77]. For the purposes of this study, these alien species have been removed from the analyses and the remaining plant species are considered as native.

### Data analysis

The elevational gradient of Crete (0–2456 m) was divided into 24 100-m vertical intervals and the species richness of each elevation interval was calculated as the total number of species of this interval [19]. All species were considered as present in every 100-m interval between their minimum and maximum elevation limits, under the assumption that species have continuous distributions [78]. Area estimation of each 100-m interval was made by 1∶50,000 digitized topographic maps of the Hellenic Mapping and Cadastral Organization, using digital elevation models in ArcGIS 3D Analyst.

Plants with restricted distribution ranges (endemics) were grouped into two categories. The first category includes Cretan or Single Island Endemic species (SIE) with a distribution range restricted to the main island of Crete. The second category, here after Subendemic species (SUBE), includes species with restricted distribution ranges to a) the Kriti-Karpathos phytogeographical area as it is defined in Flora Hellenica [79], b) the Aegean islands beyond Kriti-Karpathos phytogeographical area, c) the Aegean islands and the Greek mainland and d) the Aegean islands and Anatolia.

Species density was calculated for each elevational interval based on the following equation: *D* =  *S*/*log*(*A*), where *D* is species density, *S* the number of species and *A* the area of the elevational interval. Species densities were calculated for total , SIE and SUBE species richness.

Simple scatter plots were used to show the patterns of species richness and endemism along the elevational gradient of Crete. This was done for (1) area, (2) total, non-endemic, SIE and SUBE species richness, (3) total, SIE and SUBE species densities, (4) percentage of SIE (pSIE) and SUBE (pSUBE), (5) average elevational range of total and SIE species and (6) log-transformed values [using the formula x =  log(x+1)] of total and endemic species richness for species that occur in only one or two adjacent intervals (i.e. with elevational range 100–200 m). The elevational ranges of species belonging to this latter group are based only on observation and not interpolation, and they are used to check possible artefacts due to interpolation method [11]. Simple regressions were used to correlate patterns of species richness and endemism with elevation. The effect of elevation on pSIE and pSUBE was analyzed using generalized linear models (GLMs) fitted with a quasi-binomial family error, because our data were under-dispersed. Simple regression analyses were also used to correlate (1) total, (2) SIE and (3) SUBE species richness to log-area of each elevational interval. The elevational ranges of endemic and non-endemic species were compared using the Mann-Whitney U test. To test whether the relationship between the number of SIE and elevation was significantly unimodal, we used generalized additive models (GAM) [80].

In order to investigate the penetration of lowland SIE and SUBE to the corresponding mountain floras of the island, we used SIE and SUBE species occurring in the lower and upper one-third of the gradient. Species present in the lower one-third of the gradient were considered as lowland species and we calculated the ratio number of lowland species/number of total species, for the upper one-third of the gradient, both for SIE and SUBE. The decrease of the ratios with elevation was tested using generalized linear models (GLMs) with a quasi-binomial family error, because of under-dispersion of the data. A paired *t*-test was used to examine if ratio values of SIE and SUBE were significantly different.

For the evaluation of Rapoport's elevational rule, the correlation of mean species altitudinal ranges for all species found in each elevational interval with altitude has been examined. Then, the observed correlations were compared with the results from a fully stochastic null model for species richness gradients within a bounded domain that simulates range size and randomizes range placement within differently defined boundaries and this approach is the same as Model 3 in [8] and as is discussed in [81].

Mid-domain effect (MDE) as well as Rapoport rule have been tested using Range model 5 [82], [83], an animated graphical freeware application designed to demonstrate the mechanism behind the MDE, with tools for exploring the effects of both theoretical and empirical range size frequency distributions (RSFDs) for one-dimensional domains. Following MDE definition [81], only species ranges that are fully contained in the geographical domain under consideration have been used in the analysis. The possible underestimation of elevational range sizes for highland species, as the Cretan mountains are not high enough, is not significant for the Cretan flora since only three species (0.14% of the total flora) have their lower temperature tolerance above 2000 m. Two of them are SIE (*Myosotis solange* at 2000 m and *Nepeta sphaciotica* at 2200 m) with well studied populations and a very restricted elevational range of 100 m. In addition, only 34 species (1.9%) have their lower temperature tolerance at more than 1500 m of which 14 are SIE. Only 8 of them (0.4% of the total flora) are having their highest elevational limit above 2300 m (4 of them being SIE). These very low percentages of plants having their lower elevational presence above 2000 m and their highest elevational limit above 2300 m are not affecting MDE analysis.

Two different subsets of species have been used in the MDE analysis, i.e. total species richness (A) and SIE (B), where the ranges of the points are shown as horizontal lines centred on their midpoints. Then, a statistical evaluation of the predicted results of the fully stochastic model for the two different subsets of species richness gradients within a bounded domain mentioned above, in relation to the observed patterns has been used, as it is proposed by [81]–[83]. For any x point in the domain, richness is computed as the number of horizontal range lines that a vertical line at x (the broken line) would intersect. Correlations of species numbers predicted for the two different subsets for different elevational gradient to the ones of our database were examined. The predictive power of the MDE can be assessed using the coefficient of regression R^2^ between observed and simulated species richness values [84].

The elevational ranges of the entire local endemic floras of Crete and the Peloponnese (194 and 163 species, respectively) were used, in order to compare elevational range sizes between Crete and the Peloponnese. Data on the elevational ranges of the Peloponnesian plants were taken by [85]. A paired *t*-test was used to examine if average elevational range sizes along the elevational gradient were larger in Crete than the Peloponnese. Furthermore, in order to investigate the participation of lowland endemics to the composition of the endemic mountain floras of Crete and the Peloponnese, we used endemic species occurring in the lower and upper one-third of the gradient. We calculated the ratio number of lowland endemics/number of total endemics, for the upper one-third of the gradient, both for Crete and the Peloponnese. The decrease of the ratios with elevation was tested using generalized linear models (GLMs) with a quasi-binomial family error and a paired *t*-test was used to examine if ratio values were higher in Crete than the Peloponnese.

The composition of the two local endemic floras, however, is completely different and species-specific traits may bias the results. So, in addition to local endemic floras, two other species groups were used for the same purpose. The first group includes 19 Cretan SUBE species also occurring in the Peloponnese. Each species has been selected ensuring that it is represented in both areas by at least three subpopulations and having comparable extent of occurrence in Crete and the Peloponnese. The second group includes 14 pairs of morphologically closely related species. Each pair consists of a species that it is present in the Peloponnese (but in some cases it is also distributed beyond the Peloponnese) and a morphologically closely related species endemic to Crete (SIE) (differentiated island-mainland endemics, DIME). Each pair has been selected ensuring that its species are represented by similar number of subpopulations and they also have comparable extent of occurrence on both areas. The elevational ranges of SUBE and DIME were used in order to calculate an Island/Mainland Amplitude Ratio (IMAR) of elevational ranges [22]. Species enlarge their elevational ranges for a ratio >1 and reduce it for a ratio <1. All data underling elevational range analyses have been drawn from carefully chosen species whose native nature in Crete and the Peloponnese is beyond any doubt. We tested for differences in elevational range sizes for both SUBE and DIME in Crete and the Peloponnese by performing a regression of elevational range sizes against elevational mid-points of the species, in order to correct elevational range sizes for the observed effect of elevation. Afterwards, the residuals were compared using paired *t*-tests. An unpaired *t*-test was used to examine if IMAR values between SUBE and DIME were significantly different. Regression analyses, generalized linear models and generalized additive models were performed using the statistical package R version 2.15.2. All other analyses were performed using Statistica 7 [86].

## Results

### Elevational gradient of species richness

The dataset created using all available floristic and elevational data concerning the island of Crete includes 1825 native vascular plant species. The non-endemic element includes 1473 species (80.7% of the total flora), while the number of species per endemic category is as follows: SIE (194, 10.6%) and SUBE (158, 8.7%). The SUBE category includes phytogeographical area endemics (36, 1.9% of the total flora), Aegean endemics (37, 1.9%), Greek-Aegean endemics (60, 3.2%) and Anatolian-Aegean endemics (25, 1.3%).

**Figure 2:**
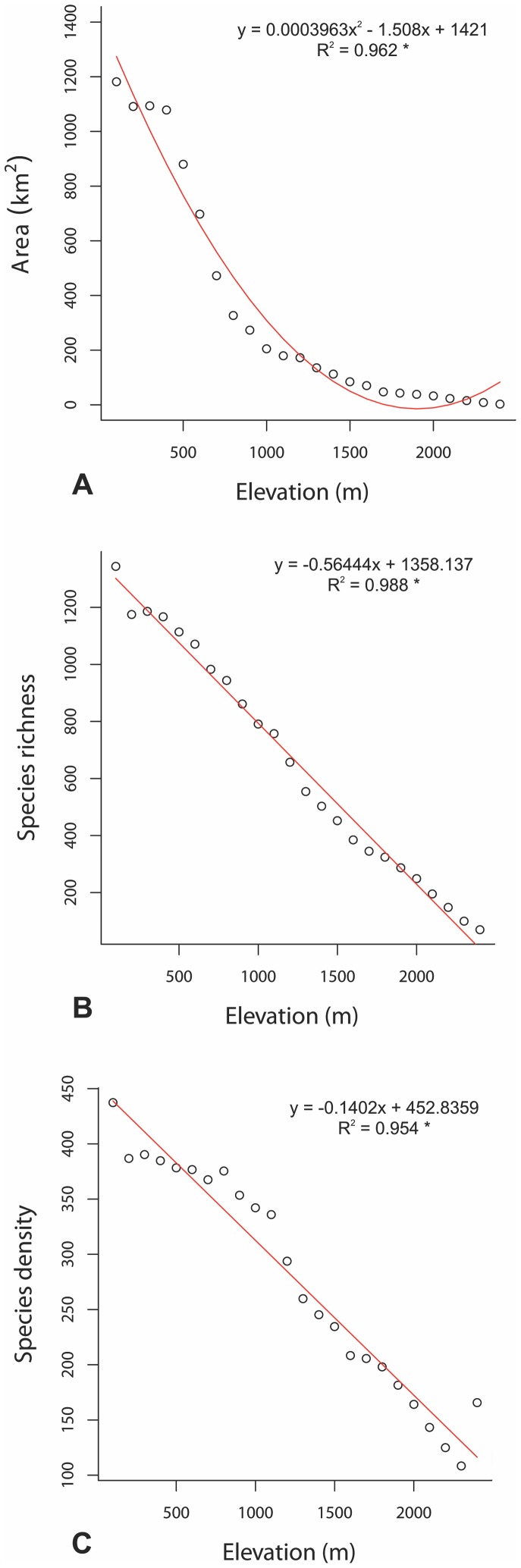
Elevational gradient of area, vascular plant species richness and density in Crete. Trend lines were set by simple regressions. (* p<0.001).

With increasing elevation, the area for each elevational interval decreases (Fig. 2a) and this reduction of area with elevation is followed by a strong monotonically decreasing trend in species richness and species density to the entire elevation range of Crete (Fig. 2b–2c). A steep decrease exists between 100 and 200 m for species richness, followed by a small plateau between 200 and 400 m. From 500 to 2400 m a continuous and rather uniform decrease of species richness is observed. The non-endemic species richness shows a similar elevation pattern with somewhat steeper decrease of species richness with elevation (graph not reproduced here). There is a strong positive correlation between log-area of each elevational interval and total species richness of this interval (R^2^ = 0.935, p<0.001). This strong positive correlation also exists between area and all SUBE groups (R^2^>0.90, p<0.001). The correlation is weaker for SIE (R^2^ = 0.325, p<0.01).

### Elevational gradient of endemism

**Figure 3:**
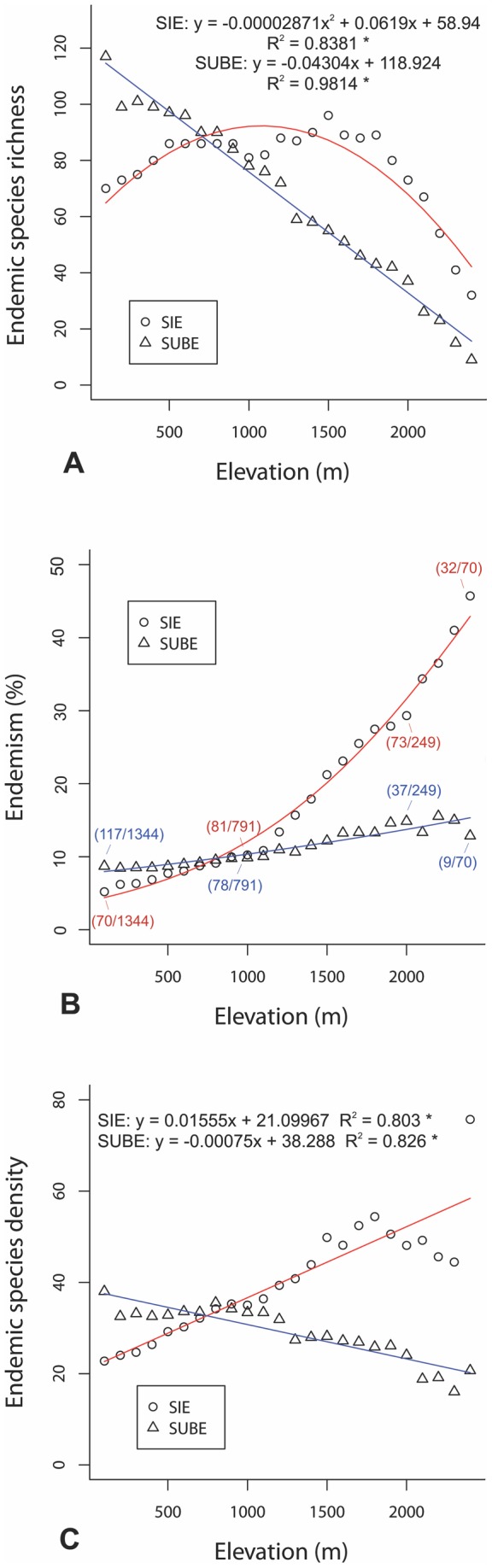
Elevational gradient of vascular plant endemism in Crete. Elevational gradient of (A) endemic species richness, (B) percentage of endemic species (with species numbers provided for 100, 1000, 2000 and 2400 m elevational intervals) and (C) endemic species density, for Single Island Endemics (circles) and Subendemics (triangles). Trend lines were set by simple regressions (A, C) and generalized linear models (B). (* p<0.001).

**Figure 4:**
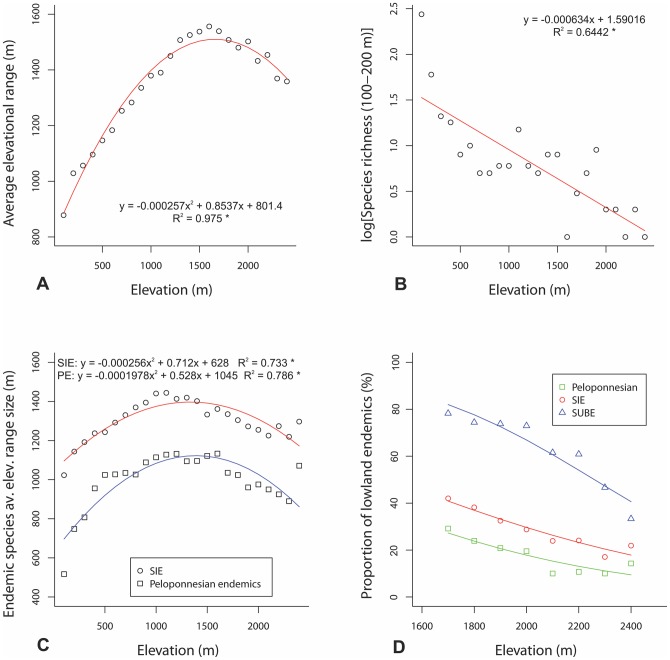
Data on the elevational range size of the vascular plants. (A) average elevational range of total species, (B) log-transformed total number of species with a very narrow elevational range (100–200 m), (C) average elevational range of Cretan endemics (circles) and Peloponnesian endemics (squares) and (D) proportion of lowland species in the upper one-third of the elevational gradient for Cretan subendemics (triangles), Cretan endemics (circles) and Peloponnesian endemics (squares). Trend lines were set by simple regressions (A, B, C) and generalized linear models (D). (* p<0.001).

In contrast to overall and non-endemic species richness, SIE show a significantly unimodal response (p<0.001) to elevation gradient with a peak at 1500 m (Fig. 3a). On the contrary, the number of SUBE shows a monotonic decrease along the elevational gradient of Crete (Fig. 3a), which is rather constant in all SUBE groups. Elevation had a significant effect on pSIE (GLM: F = 1620.9, p<0.0001) and pSUBE (GLM: F = 209.14, p<0.0001). Although both pSIE and pSUBE increased with elevation, that of pSIE was more intense (slope: 0.016) compared to pSUBE (slope: 0.00003) (Fig. 3b). SUBE species density show a monotonic decrease with increasing altitude, while SIE species density increases monotonically from sea level to 1800 m a.s.l. and then it slightly decreases upwards (Fig. 3c). A significant decrease of the ratio lowland species/total species in the upper one-third of the gradient was identified by the generalized linear models, for both SUBE (F = 53.67, p<0.001) and SIE (F = 58.77, p<0.001) (Fig. 4d). The ratio values for SUBE were significantly higher than that of SIE (paired *t*-test: *t* = −9.6, d.f.  = 7, p<0.001).

### Elevational range size

Elevational range profiles of the vascular plants of Crete showed that most species occupied wide elevational ranges along the gradient; 788 species (43.2%) had elevational ranges ≥1000 m, while 643 species (35.2%) ≤500 m. Although the distributions of total endemics and non-endemic species elevational ranges were not significantly different (Mann-Whitney U = 71, *n*_endemic_  = 351, *n*_non-endemic_  = 1474, p = 0.7597), SIE have an average elevational range of 972 m and SUBE of 991 m (phytogeographical area endemics 1122 m, Aegean endemics 884 m, Greek-Aegean endemics 945 m and Anatolian-Aegean endemics 1072 m), while the non-endemic species have an average elevation range of 830 m. The average elevational range of total species increases from sea level to 1600 m and then it slightly decreases up to 2400 m (Fig. 4a). Number of species with very narrow elevational range (100–200 m) decreases with increasing elevation (Fig. 4b). The average elevational range of SIE (Fig. 4c) follows a similar pattern with that of total species richness, with the difference that wider elevational ranges are observed in lower intervals (1000–1100 m).

The twenty one percent of the Cretan flora (381 species) represent species distributed in only one or two elevational intervals from which only 15% (57 species) are strictly littoral species. All endemic categories are underrepresented in this group, which is mainly composed of common plants or weeds with wide elevational ranges elsewhere, wetland plants and relict species with disjunct distribution patterns in the east Mediterranean area. Species diversity of narrow range species is abruptly decreased from sea level to 200 m and then they remain relatively stable to very low level until 2400 m (Fig. 4b). This clear pattern does not change even if all littoral species are removed from the analyses.

### MDE and elevational Rapoport effect

Regarding Rapoport's rule, the observed ranges of altitudinal distribution for the species of total flora as well as of SIE show a significant correlation with the predicted ones from a null model simulation through Range model 5.0 (R^2^ = 0.434, p<0.001 and R^2^ = 0.403, p<0.001, respectively), as also for the SUBE (R^2^ = 0.397, p<0.001) and the non-endemics (R^2^ = 0.295, p<0.001).

**Figure 5:**
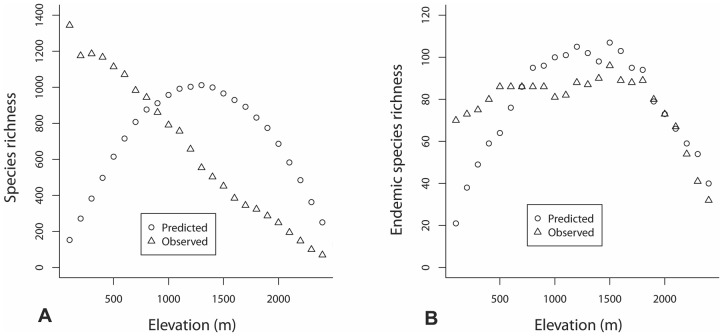
Predicted of the null model simulation and observed values of species richness concerning mid-domain effect. The comparisons concern to (A) vascular plant species richness and (B) Single Island Endemics.

The comparison of species richness curves for the different species subsets used in this study with the predicted species distribution patterns resulted by the simulations realized through Range model for the different elevational gradients following MDE, is presented in Fig. 5. Values resulted by the simulations for the SIE and SUBE seem to be significantly correlated with the observed species richness (R^2^ = 0.566, p<0.001 and R^2^ = 0.250, p<0.001, respectively). There is a not significant correlation as well as MDE's explanatory power concerning species richness patterns of total and non-endemic Cretan flora.

### Elevational range expansion of Cretan plants

Both Cretan and Peloponnesian [85] endemic plants show a unimodal response to elevation gradient. The average elevational range of Cretan endemics (972 m), however, is higher than that of the Peloponnesian endemics (658 m). Furthermore, the average elevational ranges of Cretan and Peloponnesian endemics along the elevational gradient of Crete and the Peloponnese, respectively, are significantly different (paired *t*-test: *t* = −23.5, d.f.  = 23, p<0.001) (Fig. 4c). Although the trend lines are almost identical with a peak at middle altitudes, the average elevational range of the Peloponnesian endemics is invariably lower than that of Cretan endemics. Elevational range expansion of Cretan plants is evidently higher for lowland than highland endemics. Lowland SIE that start their elevational distribution from sea level (*n* = 66) have an average elevational range equal to 1078 m, while the corresponding value for the Peloponnesian endemics (*n* = 43) is 556 m. Highland SIE that start their elevational distribution above 1000 m a.s.l. (*n* = 56) have an average elevational range of 752 m, while highland Peloponnesian endemics (*n* = 49) have an average elevational range of 551 m. As a result, the ratio lowland endemics/total endemics is significantly higher (paired *t*-test: *t* = 11.0, d.f.  = 7, p<0.001) in Cretan than in the Peloponnesian upper one-third of the gradient (Fig. 4d). Thus, the endemic mountain flora of Crete is more efficiently enriched by lowland species than the endemic mountain flora of the Peloponnese. As with SUBE and SIE, a significant (F = 19.58, p<0.005) decrease of the ratio lowland species/total species in the upper one-third of the gradient was also identified by the generalized linear models for the Peloponnesian endemics.

If the insular syndrome of niche enlargement has not affected the elevational range of Cretan plants, we should expect the IMAR of SUBE and DIME in Crete and the Peloponnese to average ∼1.0. Elevational ranges of SUBE were significantly higher in Crete than the Peloponnese (paired *t*-test: *t* = 3.44, d.f.  = 18, p<0.005). In fact, of the 19 SUBE considered, 10 (52.6%) gave an IMAR value >1.0, six (31.6%) gave an IMAR <1.0, while three species (15.8%) gave an IMAR value ∼ 1.0 (Table S1). The dominance of range expansions also characterize the DIME, since out of the 14 species considered, eight (57.1%) gave an IMAR value >1.0, four (28.6%) <1.0 and two species gave an IMAR value ∼ 1.0 (Table S2). Elevational ranges of DIME between Crete and the Peloponnese, however, were not significantly different (paired *t*-test: *t* = 1.63, d.f.  = 13, p = 0.126). The average IMAR values for SUBE and DIME are 1.26 and 1.41, respectively (Table 2), but the IMAR values between them were not significantly different (unpaired *t*-test: *t* = −1.24, d.f.  = 14, p = 0.238). Elevational range expansions of Cretan plants resulted in SUBE either from raising the upper limits of the island species compared with the mainland (five species), from lowering their lower limits (one), or both (four). For DIME raising the upper elevational limits seems to be the rule for the observed elevational range expansions of Cretan plants.

**Table 2:** Elevational ranges of subendemic species occurring both in Crete and the Peloponnese (SUBE) and differentiated island-mainland endemics (DIME) in Crete and the Peloponnese.

Categories	IER	MER	IMAR	*n*
SUBE	1347±614	1158±457	1.26±0.58	19
DIME	1214±604	964±497	1.41±0.61	14

Average island elevational range (IER), average mainland elevational range (MER) and average island/mainland amplitude ratios (IMAR) for SUBE and DIME (results ± 1 SD).

## Discussion

### Elevational gradient of species richness

Species richness-elevation relationships have received considerable attention during the last two decades, as a response to the major challenge of documentation and explanation of global and regional gradients of species richness [2], [4], [87]. For vascular plants, most studies indicate hump-shaped relationships between species richness and elevation, e.g. [11], [25], [26], while some studies suggest that species richness decreases monotonically with elevation, e.g. [31], [32]. The comparability of the results, however, is often affected by potential biases, such as incompletely sampled gradients, differences in regional areal size, sampling method and taxon specific traits [4]. In this study, species richness of vascular plants showed a clear monotonically decreasing pattern across the entire elevational gradient of Crete. Elevational species density pattern further confirmed this trend, despite the use of range interpolation method that itself can amplify mid-elevation richness humps, i.e. overestimation of species richness in mid-elevation intervals compared to the extremes of the gradient [78], [81]. Other studies from Crete also reported similar elevational richness pattern for terrestrial isopods and ground spiders [88]–[90], suggesting that this is a more universal pattern on the island. Bat species richness, however, was not significantly affected by elevation in the same area [91].

Our results are fully consistent with the evidence on the geodynamic evolution of Crete, as it seems that a true mountain flora was never existed on the island. Only 34 vascular plant species of Crete are high-elevation specialists. The mountain flora of Crete is mainly composed of lowland species that were able to colonize the newly formed mountains. Thus, the monotonic decline of vascular plant species richness and density observed along the entire elevational gradient of Crete is probably influenced by the absence of a true mountain flora and the colonization of Cretan mountains mainly by lowland species that were able to withstand the harsh climatic conditions of the mountains. The high-altitude fauna of Crete has also been derived from tolerant species of the lowlands [90], [92], [93]. An efficient enrichment of Cretan mountains by long-distance dispersal is unlikely, since reduced species richness with increasing elevation on islands have also been linked to the declining number of arriving colonists (propagule pressure) to highland compared to lowland areas [36]. Cretan lowlands (Fig. 1) are much larger target for propagules than Cretan highlands, and the same is true for most islands worldwide. Highland areas on islands are more effectively isolated than lowlands, as comparable environmental conditions on neighboring islands or the continent are further apart, smaller in area and lower altitude ecosystems act as barriers [36], [94]. Beyond its impact on the Cretan mountain flora, post-isolation uplift of the Cretan mountains must have affected the lowland flora of Crete by forming new biotic barriers and profoundly changing the hydrology and climate of the island.

The low altitude peak of species richness on Crete may have also been further supported by human transferred species, since human activities are mainly confined to lowland areas. Plant species already introduced in prehistoric or early historic times may be perfectly integrated into the native plant communities and it may be extremely difficult, or even impossible, to distinguish them from the truly native ones [60].

Area is a principal factor in all species richness analyses [39], [95] and it should be a main component of studies examining species richness along elevational gradients, because the area extent in different elevational intervals usually varies greatly. The influence of area as a determining factor for regional species richness patterns along altitudinal ranges has been shown for different taxa [31], [96], [97]. In Crete, area alone explains 93.5% of the variance of species richness among the elevational intervals and it also explains more than 90% of the variance in all endemic species' categories, except that of SIE. These results indicate that area may be the main causal factor for the observed pattern of species richness, with respect to the correlation of area *per se* with habitat diversity [98]. This pattern is largely in accordance with the prediction of [19] that species richness of elevational intervals should vary directly with their total area, peaking in those intervals that cover the largest area. In our study, however, the monotonically decreasing pattern was also identified for species density, indicating that this pattern is not an artefact of area. This species density pattern is in contrast with the prediction of [19], whereby species density should peak at an intermediate elevation and the peak should occur at a transition zone between the species-rich, juxtaposed communities.

### Elevational gradient of endemism

Contrary to the total species richness-altitude relationships that have been studied extensively [3], [4], [5], [25], [26], [31], [32], [78], [87], endemic species richness-altitude relationships have received much less attention. It has been documented that endemic species richness usually peaks at higher elevation than total species richness, as a result of the increasing isolation and decreasing surface area of high mountain regions, leading to small, fragmented species populations that are prone to speciation ([15] and references therein). On oceanic or long isolated continental islands like Crete, however, mountain isolation is not the main isolating factor. Island isolation is expected to affect endemic species richness, and probably its relationship to altitude, in a rather complex way. In this study, SIE species richness showed a unimodal response to elevational gradient with a peak at 1500 m a.s.l. This unimodal pattern for SIE is holding steady when individual mountains of Crete are considered, indicating that the hump-shaped pattern of SIE is not an artefact of increasing heterogeneity of the SIE species composition with increasing altitude among the Cretan mountains. SUBE species richness, however, show a monotonic decrease along the elevational gradient. These results are further supported by endemic species densities along the elevational gradient and they are obviously not affected by taxon-specific traits, since numerous plant families and genera are represented in all endemic species groups averaging taxon-specific patterns.

pSIE is a strong indicator of diversification rate [99] and its increase with elevation has been documented for oceanic islands [36]. In continental islands, however, the effective separation of passive (relictual) from active endemism often faces insurmountable obstacles. High elevation ecosystems of Crete might serve either as refugia for old high mountain species that were able to withstand climatic fluctuations during the Pleistocene [49], [94] or as diversification cradles because of increased geographic and ecological isolation. The recent uplift of the Cretan mountains, however, indicates that the relict element of the Cretan flora should mainly consist of lowland species. Increased species impoverishment with increasing elevation has created the conditions under which the colonization of the Cretan mountains by lowland plants took place [49].

In this study, the observed differences between pSIE and pSUBE elevational gradient in Crete raise a question about the underlying mechanisms. The relict character of SUBE species in Crete is supported by (1) their present distribution range, which pass over well-established long-standing sea isolation barriers, (2) their elevational distribution pattern on Crete (90% of them are present at the lower one-third of the gradient), (3) their large elevational range sizes on Crete (the largest among all species groups examined) and (4) the lack of evident adaptations to promote efficient long-distance seed dispersion in c. 85% of them. As the proportion of the non-differentiated, mainly relictual SUBE species is relatively stable along the elevational gradient of Crete, the increased pSIE with increasing elevation is an indication of intensified diversification processes towards Cretan mountains. Moving upwards following the uplift of the Cretan mountains, lowland Cretan plants have subjected increased selective pressure from their changing environment. Elevational range expansions on islands in most cases accompanied morphological differentiations of insular populations. So, they seem to require genetic changes [22]. The strong correlation between maximum island elevation and pSIE on the Aegean Islands identified for neo-endemic species [100] support the promotion of diversification by elevation-driven ecological isolation [36], [94]. Our results indicate that the increase of pSIE with increasing elevation is possibly the result of diversification processes towards Cretan mountains. But in fact, from the 95 SIE distributed on the upper one-third of the elevational gradient of Crete only 10 species (10.5%) are high-elevation specialists. Thus, diversification processes were probably more intensive in the SIE species rich mid-elevation intervals and the increased pSIE values at high altitudes are due to the combined effect of colonization by low- and mid-elevation SIE and the reduced species richness at high altitudes. These results are also congruent to the Pleistocene age of the upper mountain zone of Crete. Several lowland relatives of Cretan mountain endemics cannot be located today, probably because they have been extinct during relaxation processes after island isolation and human disturbances mainly confined to lowland areas. Increased human impacts in Cretan lowlands can hardly be seen as the main reason for the increase of SIE with elevation, as there is no reason to believe that SIE are differently affected by human pressure than SUBE.

Are there other reasons that pSIE increases with elevation on Crete, while pSUBE does not? An explanation could be found on the increased extinction rates of plant species with increasing elevation on the mainland after island isolation vs. the maintenance of their populations on the Cretan mountains, turning them to Cretan endemics (SIE). The result of this scenario should be an increased number of relictual SIE in the Cretan mountains, which is contrary to the recent uplift of the Cretan mountains and the decreasing number of the mainly relictual SUBE with increasing elevation. Another explanation could be found on the influence of the enlarged elevational range sizes of SIE towards Cretan mountains, which together with the decreasing species richness with increasing altitude on Crete, create increased pSIE values with increasing elevation. SUBE, however, have the largest elevational ranges among all species groups examined. Furthermore, the participation of lowland species to the composition of the mountain flora is significantly higher in SUBE than in SIE (Fig. 4d). This means that the increased pSIE values with increasing elevation in Crete is not an artefact of the enrichment of high elevation areas by lowland SIE.

The results of this study indicate a significant role of mountain areas (especially mid-elevation areas) in promoting diversification processes of Cretan plants, but further work could address these issues. Applying robust phylogenetic analyses for the vascular endemic plants of Crete would clearly allow for a better evaluation of how historical and evolutionary factors influence endemic species richness.

### Elevational range size

A positive correlation between geographical distribution range and elevational range of plant species is expected, since species with wide elevational range should have increased ability to compete with other species from different vegetation zones and to pass over ecological barriers (e.g. mountains, unsuitable habitats) and distribute over large areas. This is true for continental areas, where species can expand their geographical ranges without sea barrier limitations; but on oceanic or long isolated continental islands, like Crete, historical factors (i.e. colonization time) may also influence this relation. The search for mechanistic explanations for macroecological patterns may be inevitably constrained significantly by the idiosyncratic nature of historical events [101].

Surprisingly, widely distributed species (non-endemics), in Crete, have smaller elevational ranges than geographically more restricted species (endemics). Long-distance migration is expected to have enriched the non-endemic flora of Crete after its isolation. Furthermore, oceanic and long-isolated continental islands support a low diversity of native species combined with high endemism, and they are often perceived as highly susceptible to invasions [102]–[104]. Compared to neighboring mainland areas, Mediterranean island floras have a significantly higher proportion of alien plant species [105]. The higher vulnerability of islands relative to comparable continental areas has been attributed to proportionally lower native diversity, the existence of unsaturated communities, lower competitive ability of native species and the higher susceptibility of insular species to the ecological impacts of the invaders [106]. Therefore, the Cretan paradox of small elevational ranges for non-endemic Cretan species is probably a result of short-lasting presence on the island for many of these species. They are “recent” colonists, or invaders difficultly distinguished from the truly native species. They have not had adequate time to colonize their potential wide elevational range until today. Another explanation of this pattern includes the subsequent reduction of fitness of initially superior invaders, which at first become widespread and abundant, but as they evolve become progressively rarer and more restricted, embarking an evolutionary trajectory that leads ultimately even to extinction and replacement by another wave of colonists [107]. On the contrary, endemic plants are well-adapted to the local environmental conditions, they have experienced Pleistocene climatic fluctuations on Crete and they are present on the island for an adequate lapse of time to distribute over a large elevational range. Moreover, strong climatic fluctuations act as an evolutionary filter for climate-specialized species, thus favoring pre-existing of climate-generalist species, usually of wider elevational ranges [108].

### MDE and elevational Rapoport effect

Our results indicate that MDE is not the main underlying mechanism of the elevational gradient of vascular plant species richness in Crete. MDE has a well explanatory power on plant diversity patterns only for the pool of SIE, a low explanatory power on SUBE species richness patterns and it did not significantly explain species richness patterns of total and non-endemic Cretan flora. The very good correlation between the observed and the predicted (by MDE) elevational species richness patterns for SIE and the high value of the coefficient of determination R^2^ could be attributed to the increased elevational ranges of SIE, leading to increased overlaps of elevational ranges at mid-elevation intervals. SUBE elevational species richness pattern, however, have a significant correlation to the predicted MDE model but a low value of R^2^, although they have the largest elevational ranges among all plant species groups examined. This suggests that geometric constraints may not be the causal factor of mid-elevation peak for SIE species richness. The hump-shaped pattern of SIE species richness seems to be created by the influence of historical processes, especially speciation.

Our results only partly conform to Rapoport's elevational rule, as is also the case for other taxa in the Cretan area such as ground spiders [90]. Monotonic decrease of vascular plant species richness with increasing elevation in Crete agrees with Rapoport's elevational rule. This rule has been related to the rescue effect [6]. The possibilities for a population to be rescued on islands decreases with increasing elevation due to the combined effect of island and mountain isolation mechanisms. So, monotonic decrease of species richness is expected to be a more common pattern in island than to continental biota. Our results, however, do not fully support the predicted by Rapoport's elevational rule increase of elevational range sizes with increasing elevation.

### Elevational range expansion of Cretan plants

Cretan SIE and SUBE species have been experienced significant elevational range expansion compared to mainland species. Elevational range expansions are also known to occur in several widespread species of the Cretan flora [49]. Plant species of low- or mid-elevation areas elsewhere in the Mediterranean Basin (e.g. *Andrachne telephioides*, *Coridothymus capitatus*, *Erica manipuliflora*, *Euphorbia acanthothamnos*, *Sarcopoterium spinosum*, etc.) are distributed in Crete from sea level up to the highest mountain peaks. Unfortunately, the lack of accurate data on the elevational ranges of these species in the Peloponnese does not allow us a comprehensive comparison of the whole floras.

Our results on elevational range expansions of Cretan plants are largely congruent to the post-isolation uplift of the Cretan mountains and their occupation mainly by pre-existing lowland species. Ecological release triggered by low competition from mountain species has led to elevational range expansion of Cretan plants. In several cases, environmental filtering towards Cretan mountains has not led to diversification, i.e. the case of SUBE species. They have been experienced significant expansion of their elevational ranges in Crete compared to mainland areas, but their number decrease monotonically with increasing altitude. Only few high-elevation specialists exist within this group and the weakly increased pSUBE towards Cretan mountains is mainly supported by the elevational range expansion of lowland species.

The comparison of elevational range sizes between Cretan and Peloponnesian endemic plants (DIME), as well as between SUBE distributed both in Crete and the Peloponnese, also support the elevational range expansion of Cretan plants. Although, SIE reach higher IMAR values than SUBE, their elevational range expansion was not significant. The difference between SUBE and DIME IMAR values was also not significant. There is no doubt that the present distribution ranges of the species used in these analyses are of a relict nature. Thus, our results indicate that the realized genetic changes of the differentiated species (SIE) do not make them more efficient in order to enlarge their fundamental niches. Elevational range expansions for both SIE and SUBE were mainly towards Cretan mountains and this result is further supported by the increased participation of lowland species to the composition of the corresponding mountain floras.

## Conclusions

Our results add valuable insights for an improved understanding of elevational species richness and endemism patterns on continental island systems. The impact of the post-isolation mountain uplift to the present elevational species richness and endemism patterns on a continental island has been studied for the first time. We found that total and SUBE vascular plant species richness monotonically decreases with increasing elevation in Crete, while SIE show a unimodal response to elevational gradient. These results are congruent with the post-isolation uplift of the Cretan mountains and their colonization mainly by the available lowland vascular plant species. Moving upwards, following mountain uplift, the Cretan flora was impoverished as a result of environmental filtering and the mountain areas have been mainly colonized by tolerant lowland species. As a result of island and elevation-driven ecological isolation synergy, Cretan highlands are more effectively isolated than Cretan lowlands, resulting to increased species impoverishment with increasing elevation. The influence of species richness-area relationships, probably together with long-distance dispersal and species transferred by humans, has further supported this pattern. Both, endemism generated by extinction and *in situ* evolution are expected to contribute to the present endemic flora composition, but the increase of pSIE with increasing elevation in Crete can only be regarded as a result of increased diversification processes towards Cretan mountains. Relict endemics are expected to concentrate mostly in low- to mid-elevation intervals, as it is indicated by the post-isolation uplift of the Cretan mountains and the elevational distribution patterns of SUBE species.

Our results also support the elevational range expansion of the Cretan SIE and SUBE species compared to the continental control area. Tolerant plant species able to withstand the harsh climatic conditions with increasing elevation had to face reduced inter-specific competition. Ecological release triggered by increased species impoverishment with increasing altitude has led to elevational range expansion of Cretan plants.

Growing body of evidence supports the influence of mid-domain effect on elevational species richness patterns. MDE, however, is not the underlying mechanism of vascular plant species richness elevational gradient in Crete, indicating that the idiosyncratic nature of historical events can strongly influence elevational patterns of species richness on continental island systems. Elevational gradient of vascular plant species richness in Crete are congruent with Rapoport's elevational rule, but elevational range sizes of Cretan plants only partly conform to its predictions.

The whole Aegean archipelago, including Cretan area, has a complex geological history and our results for the island of Crete indicate that there is not only one overriding factor that defines the response of plant species to environmental gradients on continental island systems. A corresponding combination of factors, each one with different intensity and duration of influence depending largely on historical parameters, determines elevational patterns of plant species richness and endemism in Crete.

## Supporting Information

Table S1 Elevational ranges of subendemic species occurring both in Crete and the Peloponnese (SUBE) and the resulted IMAR for each species.
(DOCX)

Table S2 Elevational ranges of the differentiated island-mainland endemics (DIME) distributed in Crete and the Peloponnese and the resulted IMAR for each species.
(DOCX)
