# Supplementary material for: Elevational Gradient of Vascular Plant Species Richness and Endemism in Crete – The Effect of Post-Isolation Mountain Uplift on a Continental Island System
Source: PLoS One. 2013 Mar 12;8(3):e59425. doi: 10.1371/journal.pone.0059425 (PMC3595250; doi:10.1371/journal.pone.0059425)
Supplement: Table S1 — Elevational ranges of subendemic species occurring both in Crete and the Peloponnese (SUBE) and the resulted IMAR for each species. (DOCX) [file pone.0059425.s001.docx]

**Table S1.** Elevational ranges of subendemic species occurring both in Crete and the Peloponnese (SUBE) and the resulted IMAR for each species.

| **Taxon** | **Crete** | | | **Peloponnese** | | | **IMAR** |
| --- | --- | --- | --- | --- | --- | --- | --- |
|  | **min alt (m)** | **max alt (m)** | **alt range (m)** | **min alt (m)** | **max alt (m)** | **alt range (m)** |  |
| *Avenulla agropyroides* | 0 | 1800 | 1800 | 0 | 1600 | 1600 | 1.13 |
| *Campanula aizoides* | 1800 | 2400 | 600 | 1200 | 2100 | 900 | 0.67 |
| *Crocus boryi* | 100 | 600 | 500 | 0 | 1500 | 1500 | 0.33 |
| *Crocus laevigatus* | 0 | 2400 | 2400 | 0 | 1100 | 1100 | 2.18 |
| *Gypsoplila nana* | 1500 | 2400 | 900 | 1100 | 2300 | 1200 | 0.75 |
| *Helianthemum hymmetium* | 1400 | 2300 | 900 | 800 | 2100 | 1300 | 0.69 |
| *Lysimachia serpyllifolia* | 400 | 2200 | 1800 | 1400 | 2300 | 900 | 2.00 |
| *Melilotus graecus* | 0 | 500 | 500 | 0 | 1000 | 1000 | 0.50 |
| *Melica rectiflora* | 0 | 2300 | 2300 | 0 | 1200 | 1200 | 1.92 |
| *Onosma erecta* subsp*. erecta* | 0 | 2000 | 2000 | 400 | 1900 | 1500 | 1.33 |
| *Orchis boryi* | 0 | 1200 | 1200 | 200 | 1100 | 900 | 1.33 |
| *Petrorhagia illyrica* subsp*. taygetea* | 300 | 2200 | 1900 | 300 | 2100 | 1800 | 1.06 |
| *Phlomis cretica* | 0 | 1500 | 1500 | 0 | 1400 | 1400 | 1.07 |
| *Ptilostemon gnaphaloides*  subsp. *pseudofruticosus* | 0 | 500 | 500 | 0 | 400 | 400 | 1.25 |
| *Ranunculus subhomophyllus* | 1100 | 2300 | 1200 | 1600 | 2200 | 600 | 2.00 |
| *Sedum laconicum* subsp*. laconicum* | 700 | 2400 | 1700 | 0 | 2200 | 2200 | 0.77 |
| *Sedum tristriatum* | 600 | 2300 | 1700 | 900 | 2200 | 1300 | 1.31 |
| *Symphytum creticum* | 0 | 1300 | 1300 | 0 | 600 | 600 | 2.17 |
| *Veronica thymifolia* | 1500 | 2400 | 900 | 1600 | 2200 | 600 | 1.50 |
| **Average IMAR** | | | | | | | **1.26** |
