# Supplementary material for: Elevational Gradient of Vascular Plant Species Richness and Endemism in Crete – The Effect of Post-Isolation Mountain Uplift on a Continental Island System
Source: PLoS One. 2013 Mar 12;8(3):e59425. doi: 10.1371/journal.pone.0059425 (PMC3595250; doi:10.1371/journal.pone.0059425)
Supplement: Table S2 — Elevational ranges of the differentiated island-mainland endemics (DIME) distributed in Crete and the Peloponnese and the resulted IMAR for each species. (DOCX) [file pone.0059425.s002.docx]

**Table S2.** Elevational ranges of the differentiated island-mainland endemics (DIME) distributed in Crete and the Peloponnese and the resulted IMAR for each species.

| **Cretan taxon** | **min alt (m)** | **max alt (m)** | **alt range (m)** | **Peloponnesian taxon** | **min alt (m)** | **max alt (m)** | **alt range (m)** | **IMAR** |
| --- | --- | --- | --- | --- | --- | --- | --- | --- |
| *Aethionema saxatile* subsp*. creticum* | 200 | 2200 | 2000 | *Aethionema saxatile* subsp*. graecum* | 200 | 1400 | 1200 | 1.67 |
| *Allium circinnatum* subsp*. circinnatum* | 0 | 500 | 500 | *Allium circinnatum* subsp*. peloponnesiacum* | 0 | 200 | 200 | 2.50 |
| *Asperula rigida* | 0 | 2100 | 2100 | *Asperula elonea* | 0 | 1200 | 1200 | 1.75 |
| *Astragalus idaeus* | 1800 | 2100 | 300 | *Astragalus agraniotii* | 1800 | 2000 | 200 | 1.50 |
| *Biarum tenuifolium* subsp*. idomenaeum* | 0 | 1500 | 1500 | *Biarum tenuifolium* subsp*. abbreviatum* | 0 | 1400 | 1400 | 1.07 |
| *Brassica cretica* subsp*. cretica* | 0 | 1500 | 1500 | *Brassica cretica* subsp*. laconica* | 100 | 700 | 600 | 2.50 |
| *Crepis sibthorpiana* | 1000 | 2400 | 1400 | *Crepis heldreichiana* | 1400 | 2300 | 900 | 1.56 |
| *Crocus sieberi* subsp*. sieberi* | 1000 | 2400 | 1400 | *Crocus sieberi* subsp*. nivalis* | 1100 | 2400 | 1300 | 1.08 |
| *Ebenus cretica* | 0 | 1300 | 1300 | *Ebenus sibthorpii* | 0 | 800 | 800 | 1.63 |
| *Geocaryum creticum* | 1300 | 2100 | 800 | *Geocaryum peloponnesiacum* | 1400 | 2300 | 900 | 0.89 |
| *Klasea cretica* | 200 | 600 | 400 | *Klasea moreana* | 300 | 900 | 600 | 0.67 |
| *Noccaea cretica* | 1400 | 2400 | 1000 | *Noccaea graeca* | 400 | 2300 | 1900 | 0.53 |
| *Odontites linkii* subsp*. cretica* | 0 | 1900 | 1900 | *Odontites linkii* subsp*. linkii* | 0 | 1200 | 1200 | 1.58 |
| *Petrorhagia dianthoides* | 0 | 700 | 700 | *Petrorhagia grandiflora* | 100 | 900 | 800 | 0.88 |
| **Average IMAR** | | | | | | | | **1.41** |
